# Prevalence of risk factors for *Clostridium difficile* associated disease in Galați

**DOI:** 10.1186/1471-2334-14-S7-P56

**Published:** 2014-10-15

**Authors:** Miruna Draganescu, Mihaela Debita, Manuela Arbune

**Affiliations:** 1 Dunărea de Jos University, Galați, Romania

## Background

*Clostridium difficile* is recognized as a major cause of nosocomial gastroenteritis with an unexpected high prevalence in the present year. Different risk factors such as previous antibiotic therapy, old age, abdominal surgery or previous proton pump inhibitors (PPI) treatment were associated with the emergence of *Clostridium difficile* associated disease (CDAD).

## Methods

Retrospective study based on medical records of patients with CDAD admitted in our hospital between June 2013 - June 2014. Diagnosis was based on clinical signs of gastroenteritis, fever and presence of *C. difficile* toxins A/B in stool (Vidas bioMerieux). Previous antibiotic treatment and duration, previous hospital exposure, abdominal surgery and PPI treatment, age and gender were considered. Statistical analysis: MedCalc.

## Results

98 patients were admitted with documented CDAD, mean age 68 (27-92) the majority 88% (86/98) were over 60 years old, 36% (35/98) men, 64% (63/98) women, 63% (62/98) were from urban area. 80% (78/98) of patients were hospitalized prior CDAD, 59% (58/98) had previous antibiotic treatment and 27.5% (27/98) had abdominal surgery. Mean hospitalization was 8.9 days (1-17) and duration over 7 days correlated with the surprisingly association of PPI in the first 3 days to vancomycin treatment (p<0.001 95% CI 21.5-32.9). The association of prior antibiotic treatment and abdominal surgery had a statistical relevance for CDAD (p=0.0349). Age over 60 years old was found to be a risk factor (p<0.0001) but no difference by gender was found (p=0.5309). No association of CDAD and urban or rural area was found.

## Conclusion

Our study confirmed the literature data regarding the risk factors of CDAD. Age over 60 year old, prior antibiotic treatment and abdominal surgery were found to be the risk factors for CDAD in our patients. Gender was not found to be a risk factor. Although we could not establish if prior PPI treatment was taken, the association of proton pump inhibitors to current vancomycin treatment was found to be a risk factor for longer hospitalization.

